# The Improvement of Dry Eye Symptoms after Pinguecula Excision and Conjunctival Autograft with Fibrin Glue

**DOI:** 10.1155/2019/6438157

**Published:** 2019-06-09

**Authors:** Jinho Jeong, Gabriel M. Rand, Taejung Kwon, Ji-Won Kwon

**Affiliations:** ^1^Department of Ophthalmology, Jeju National University College of Medicine, Jeju, Republic of Korea; ^2^Department of Ophthalmology and Visual Sciences, Montefiore Medical Center, Albert Einstein College of Medicine, New York, NY, USA; ^3^Department of Pathology, Myongji Hospital, Hanyang University College of Medicine, Goyang, Republic of Korea; ^4^Department of Ophthalmology, Myongji Hospital, Hanyang University College of Medicine, Goyang, Republic of Korea

## Abstract

**Purpose:**

To evaluate the association between pinguecula excision and subsequent improvement in dry eye syndrome.

**Methods:**

We included 30 consecutive patients with primary nasal pinguecula and dry eye symptoms undergoing ocular surgery for the first time. Criteria for pinguecula excision surgery were nasal location, yellowish color, and protrusion of conjunctiva at least 2 times thicker than adjacent normal conjunctiva as measured by anterior segment optical coherence tomography. Our primary outcomes were 3-month postoperative changes in tear film breakup time (TBUT), Schirmer test, and a dry eye symptom score.

**Results:**

30 eyes from 30 different patients (12 men and 18 women) underwent pinguecula excision and conjunctival autografting using fibrin glue. The mean age was 42.5 ± 8.35 (range 28–63) years. The preoperative protrusion ratio of pinguecula was 2.33 ± 0.28 (range 2.00–2.90). Mean preoperative TBUT, Schirmer test, and dry eye symptom scores were 5.10 ± 1.27 seconds, 6.07 ± 2.27 mm, and 2.80 ± 0.76 points. Mean postoperative 3-month TBUT, Schirmer test, and dry eye symptom scores were 7.80 ± 1.13 seconds, 7.27 ± 2.02 mm, and 0.30 ± 0.47 points, respectively. The median pre- and postoperative changes were found to be statistically significant by Wilcoxon signed-rank tests for TBUT, Schirmer test score, and dry eye symptom score.

**Conclusion:**

Surgical excision of pinguecula and conjunctival autograft using fibrin glue is an effective and safe method to improve symptoms of dry eye syndrome.

## 1. Introduction

A pinguecula is a round, yellowish, elevated fleshy tissue on the bulbar conjunctiva. It is more common in nasal limbal conjunctiva and often contains deposits of protein, fat, or calcium [1]. Although pinguecula are in most cases asymptomatic, they may cause symptoms of dryness or burning by disrupting the ocular tear film distribution, particularly when they become protruded [2].

The primary reason pinguecula is excised is for cosmesis. Argon photocoagulation has shown good results for small, nonvascularized pinguecula [3, 4], whereas surgical excision and sutureless conjunctival autografting using fibrin glue have shown good outcomes for high-grade pinguecula [5]. Grading of pinguecula is dependent on office-based assessment of pinguecula area and thickness. In the past, it was difficult to measure the thickness of pinguecula accurately, but recently, anterior segment optical coherence tomography (AS-OCT) has been used to quickly and accurately determine dimensions [6].

In our clinical practice, similar to published reports after pterygium excision, we have consistently found improvement in patients' dry eye symptoms after pinguecula excision. Interestingly, there is scant literature on the association between pinguecula and dry eye. The contribution of this study is that we examine the relationship of pinguecula and dry eye pre- and postoperatively in a quantitative manner using accurate high-resolution OCT data and changes in tear film breakup time (TBUT), Schirmer tests, and dry eye symptom scores.

## 2. Methods

**Figure 1:**
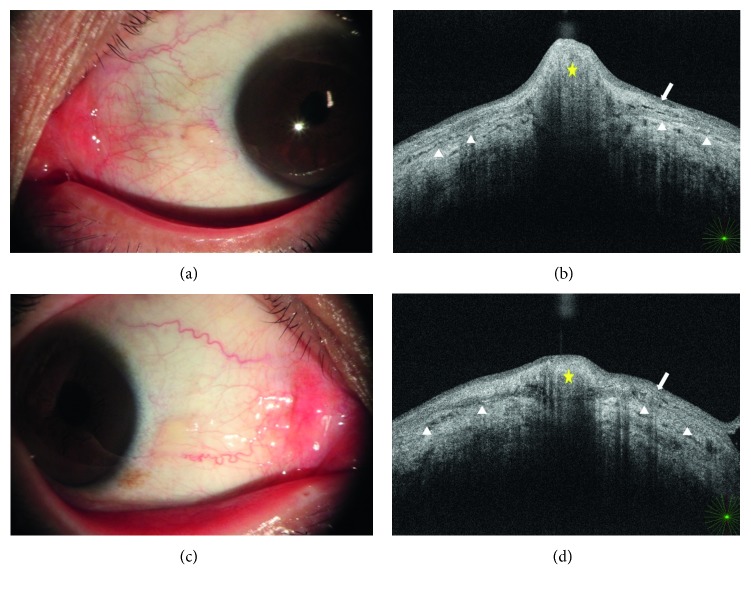
Slit lamp photograph and respective anterior segment optical coherence tomography of two patients. (a) A 27-year-old male patient with yellowish, nasal, protruded pinguecula. (b) AS-OCT of (a) showing pinguecula thickness (yellow star) more than 2 times the adjacent conjunctival thickness (distance between arrow and arrowhead). (c) A 32-year-old female patient with nasal pinguecula less protruded than that in the patient in (a). (d) AS-OCT of (c) shows pinguecula thickness less than 2 times the adjacent conjunctival thickness (distance between conjunctival epithelium (arrow) and conjunctiva-sclera junction (arrowhead)). Yellow star: pinguecula; arrow: conjunctival epithelium; arrowhead: conjunctiva-sclera junction.

This study is a retrospective chart review of patients having excision of high-grade primary pinguecula for cosmetic reasons and/or irritation. We included 30 consecutive patients meeting inclusion criteria from May 2016 to April 2017. Inclusion criteria were patients with a noninflamed, vascularized nasal pinguecula, dimensions greater than 2.0 mm × 2.0 mm, and thickness greater than 2 times that of the adjacent conjunctiva. Tissue thicknesses were measured using AS-OCT (RS-3000 Advance, Nidek Co LTD, Japan) (Figure 1). The pinguecula protrusion ratio is the thickness of the pinguecula divided by the thickness of adjacent normal conjunctiva. In cases of bilateral pinguecula, the eye with more symptoms was selected for surgery. Patients with active surface inflammation such as pingueculitis or previous ocular surface surgeries were excluded. Patients were made aware of the risks and benefits of surgery and of nonsurgical treatment modalities. We adhered to the tenets of the Declaration of Helsinki, and appropriate Institutional Review Board/Ethics Committee approvals were obtained.

All surgeries and pre-/postoperative evaluations were performed by a single surgeon (J. W. K.) using a standard technique [5]. Under topical anesthesia (0.05% proparacaine hydrochloride, Alcaine; Alcon, Ft. Worth, TX), the border of the pinguecula was first marked with gentian violet, and the outlined pinguecula was then gently excised from the underlying tenon tissue or sclera using Vannas scissors. After measuring the dimensions of the resulting defect, the superior conjunctiva was marked to the corresponding size and a free conjunctival graft was harvested. The graft was then transferred to the defective area and glued to the place using fibrin adhesives (Tisseel; Baxter, Westlake Village, CA). We excised only the pinguecula without additional margins. There was minimal tissue retraction, so graft dimensions were matched to the same dimensions of the original defect [5]. The cornea was covered with a therapeutic contact lens (Johnson and Johnson Acuvue 1 day, −0.50 diopter, 14.2 mm in diameter, 8.5 mm in base curve), and patients received levofloxacin (Cravit; Santen Pharmaceutical Company, Osaka, Japan) and 1% prednisolone acetate (Pred Forte; Allergan, Irvine, CA) eye drops four times daily for 1 week and then three times per day for 1 week. The therapeutic contact lens was removed at postoperative 1 week.

We measured preoperative and 1- and 3-month postoperative changes of TBUT, Schirmer test, and dry eye symptom scores. 3 months was considered to be a sufficient amount of time for post-op healing and prednisolone acetate drop washout. The TBUT was measured by asking the patient to look nasally and then staining the superotemporal bulbar conjunctiva with fluorescein (Fluorescein paper; Haag-Streit Diagnostics, Koeniz, Switzerland). The breakup time of the corneal tear film after one blink was recorded [7, 8]. TBUT was reported as the average of 3 consecutive measurements. The Schirmer test was performed without topical anesthesia, whereby a Schirmer strip was placed at the inferotemporal fornix, and the wet length of the strip was measured after 5 minutes [8, 9]. Patients were asked to grade cumulative ocular symptoms of dryness, stinging, foreign body sensation, and redness into one of the 5 following categories: 0 (asymptomatic); 1 (occasional symptoms but no obstacle to daily life); 2 (continuous symptoms but no obstacle to daily life); 3 (continuous symptoms with interference of daily life); 4 (continuous symptoms with interference of daily life and desiring surgical treatment). After the postoperative 1- and 3-month evaluations, patients were followed up in roughly 3-month intervals for a range of 10–15 months in order to evaluate for discomfort and/or recurrences. Statistical analyses were conducted using SPSS statistical software (version 21.0, SPSS Inc., Chicago, IL). The Wilcoxon signed-rank test was used to test preoperative and postoperative differences in our outcome variables. Linear correlations were used to assess the association of pinguecula protrusion and preoperative and postoperative changes of our outcome variables. A *p*-value of less than 0.05 was considered statistically significant.

## 3. Results

**Figure 2:**
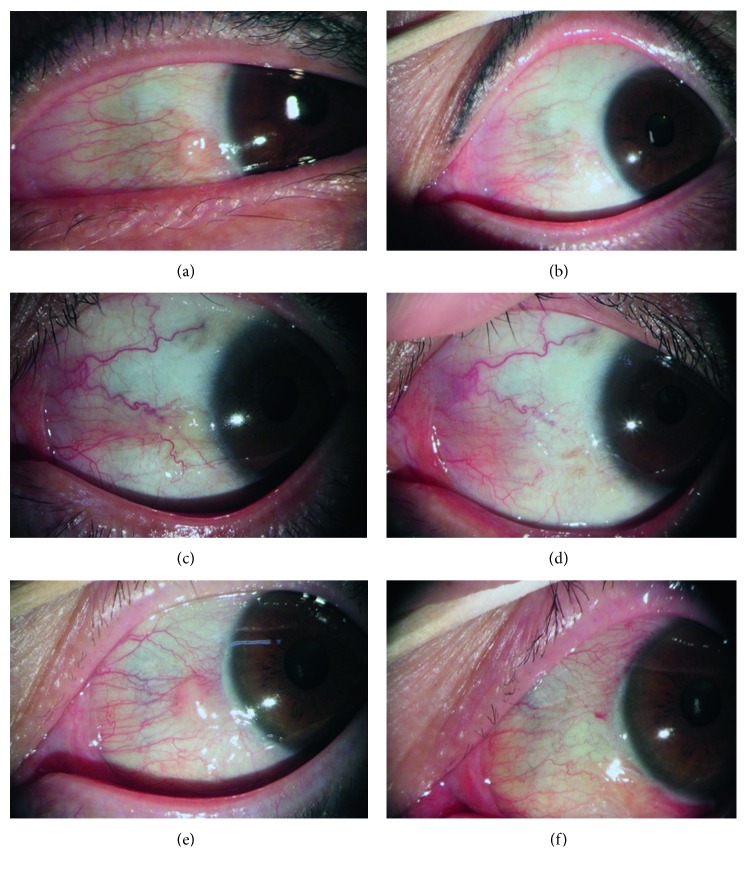
Pre- and postoperative slit-lamp biomicroscopic photographs of three patients. A 53-year-old woman with protruded nasal pinguecula (a) underwent surgery and showed vascular shrinkage and flattening of conjunctival surface; 6 months after the surgery (b). A 50-year-old man with nasal pinguecula (c) showed improvement of vascularization and conjunctival protrusion; 4 months after the surgery (d). A 48-year-old woman with prominent nasal pinguecula (e) showed improvement and restored conjunctival surface; 2 months after the surgery (f).

From May 2016 to April 2017, 30 eyes of 30 patients (12 men and 18 women) underwent pinguecula excision and conjunctival autografting using fibrin glue. Their mean age was 42.5 ± 8.35(range 28–63) years. Preoperative ophthalmic examination showed a yellowish, raised pinguecula with significant vascularization in each case and a mean protrusion ratio of 2.33 ± 0.28 (range 2.00–2.90). Histologic examination of excised tissues confirmed subepithelial solar elastosis with thinning of the overlying epithelium. The mean postoperative follow-up period was 12.27 ± 1.34 (range 10–15) months. After pinguecula excision with conjunctival autograft, it showed vascular shrinkage and flattening of the conjunctival surface (Figure 2). There was no case of recurrence during the follow-up periods. Mean preoperative TBUT, Schirmer test, and dry eye symptom score were 5.10 ± 1.27 seconds, 6.07 ± 2.27 mm, and 2.80 ± 0.76 points, respectively. At the 1-month postoperative visit, mean TBUT and Schirmer test were 7.63 ± 1.19 seconds and 6.80 ± 2.25 mm, respectively. At the 3-month postoperative visit, mean TBUT, Schirmer test, and dry eye symptom score were 7.80 ± 1.13 seconds, 7.27 ± 2.02 mm, and 0.30 ± 0.47 points, respectively. 3 months after surgery, the dry eye symptom score of 9 eyes was 1 and the remaining 21 eyes was 0. Nonparametric analyses using Wilcoxon signed-rank test confirmed statistically significant changes in the median preoperative and 3-month postoperative TBUT (sig.<0.001), Schirmer test (sig. = 0.001), and dry eye symptom score (sig. <0.001) (Tables 1 and 2).

The protrusion of the pinguecula was negatively correlated with lower TBUT (*r* = −0.552, *p*=0.002) and Schirmer test score (*r* = −0.436, *p*=0.016) and positively correlated with preoperative dry eye symptom score (*r* = 0.581, *p*=0.001) and postoperative change in dry eye symptom score (*r* = 0.504, *p*=0.005) (Table 3).

## 4. Discussion

There is a significant amount of research on the association of pterygium and dry eye syndrome [9], but very little on the association between pinguecula and dry eye, even though pinguecula is more prevalent and its pathophysiology may be different [10–12]. In this study, we found that all included measures of dry eye syndrome (TBUT, Schirmer test, and dry eye symptom score) improved after surgery. This improvement was statistically and clinically significant. There was objective 3-month improvement postoperatively in average TBUT and Schirmer test scores of 2.7 sec and 1.2 mm, respectively, and subjective improvement from symptoms on average being continuous and impacting quality of life to at worst being occasional and not bothersome.

Previous reports showed improvement in tear film stability after pinguecula and pterygium excision [2, 13–16]. It was hypothesized that the improvement in dry eye syndrome status after pinguecula surgery results from removal of the physical protrusion causing tear film instability. Most pinguecula-grading systems include quantitative measures of surface dimensions but not protrusion. We found the protrusion of the pinguecula to be statistically significantly correlated with all our dry eye outcome variables. Greater protrusion was moderately correlated with lower TBUT and Schirmer test scores. It was also moderately correlated with higher dry eye symptom scores and a greater postexcision improvement in symptoms. It follows that pinguecula protrusion may affect tear film stability and therefore TBUT, but we also found that tear film production improved postoperatively. This finding is supported by evidence in the literature of lower tear osmolarity after pterygium excision surgery [14]. Pinguecula and pterygium may influence aqueous tear production via friction against the tarsus causing local inflammation and decreasing accessory lacrimal gland secretion. Interestingly, Viso et al. published a study in which pinguecula was not associated with dry eye signs and symptoms [12]. This discrepancy is possibly due to a difference in patient populations; one including a general population and the other a population of patients with high-grade pinguecula referred to our specialty clinic.

Importantly, we found no clinically significant complications after our pinguecula excision surgeries. At a mean follow-up period of 12.3 months, there was not a single recurrence, and all patients were satisfied with their results. With respect to our surgical approach, we preferred using a conjunctival autograft with sutureless fibrin adhesive application rather than a conjunctival rotational flap or reapproximation because the latter technique often requires conjunctival sutures and may result in stretching tension to the wound. The sizes of our conjunctival autograft were relatively small; however, in instances when conjunctiva may be needed for future surgery (e.g., glaucoma filtering surgery), an amniotic membrane graft could also be considered as an alternative to our approach.

Our study has a number of limitations. The study is a retrospective analysis and was not designed to prove the causality of pinguecula excision in improving dry eye syndrome. The study does not include grading of fluorescein staining, an important measure in the study and treatment of dry eye syndrome. We attempted to control for confounders by excluding patients with active inflammation and including a prednisolone acetate washout period of more than 10 weeks. However, we did not include a nonsurgical control group and so symptom improvement may be due to a placebo effect. Lastly, the sample size was not sufficiently large to perform subgroup analyses to examine if the effect varied across different patient demographics.

In conclusion, we accurately measured the protrusion of pinguecula by comparing the relative thickness with adjacent normal conjunctiva using AS-OCT, and we found that protrusion was statistically correlated with preoperative dry eye symptom scores and that the surgical excision of symptomatic pinguecula improved not only cosmesis but also improved dry eye syndrome without any instances of recurrence or other serious complications with properly selected patients. Given the high prevalence of pinguecula [15, 16], its potential relationship to dry eye syndrome should not be overlooked, especially with significantly protruded lesions.

## Figures and Tables

**Table 1:** Clinical profile of the preoperative pinguecula patients.

	Mean	SD	Min	Max
Age (years)	42.47	8.35	28	63
M : F	12 : 18			
Protrusion ratio	2.33	0.28	2	2.9
Follow-up period (months)	12.27	1.34	10	15
Tear film breakup time (seconds)	5.10	1.27	3	7
Schirmer test (mm)	6.07	2.27	3	11
Dry eye symptom score	2.80	0.76	2	4

**Table 2:** Nonparametric Wilcoxon signed-rank test for the preoperative and 3-month postoperative change of dry eye outcome variables.

	Preoperative	Post-op 1-month	Post-op 3-month	Significance
Tear film breakup time	5.10 ± 1.27	7.63 ± 1.19	7.80 ± 1.13	<0.001
Schirmer test	6.07 ± 2.27	6.80 ± 2.25	7.27 ± 2.02	<0.001
Dry eye symptom score	2.80 ± 0.76		0.30 ± 0.47	<0.001

**Table 3:** Correlation analysis of protrusion ratio of pinguecula and dry eye outcome variables.

Pearson correlation	Protrusion ratio of pinguecula	Tear film BUT^*∗*^ (sec)	Schirmer test (mm)	Preoperative dry eye symptom score	Δ dry eye symptom score
Protrusion ratio of pinguecula	1	−0.552 (*p*=0.002)	−0.436 (*p*=0.016)	0.581 (*p*=0.001)	−0.504 (*p*=0.005)
Tear film BUT^*∗*^ (sec)	−0.552 (*p*=0.002)	1	0.691 (*p* < 0.001)	−0.407 (*p*=0.026)	0.465 (*p*=0.010)
Schirmer test (mm)	−0.436 (*p*=0.016)	0.691 (*p* < 0.001)	1	−0.231 (*p*=0.219)	−0.270 (*p*=0.149)
Preoperative dry eye symptom score	0.581 (*p*=0.001)	−0.407 (*p*=0.026)	−0.231 (*p*=0.219)	1	0.806 (*p* < 0.001)
Δ dry eye symptom score	0.504 (*p*=0.005)	−0.465 (*p*=0.010)	−0.270 (*p*=0.149)	0.806 (*p* < 0.001)	1

## Data Availability

The patient data used to support the findings of this study are available from the corresponding author upon request.
